# Supplementary material for: Nutritional care for children with feeding difficulties and disabilities: A scoping review
Source: PLOS Glob Public Health. 2023 Mar 17;3(3):e0001130. doi: 10.1371/journal.pgph.0001130 (PMC10022789; doi:10.1371/journal.pgph.0001130)
Supplement: Attachment — Submitted filename: Response to reviewers.docx [file pgph.0001130.s002.docx]

JSI Research & Training Institute, Inc.	2733 Crystal Drive	703 528 ⬝ 7474	Voice

4^th^ Floor	703 528 ⬝ 7480	Fax

Arlington ⬝ Virginia	[jsinfo@jsi.com](mailto:jsinfo@jsi.com)	Email	22202 USA	www.jsi.com

January 19, 2023

Dr. Catherine Kyobutungi, Editor-in-Chief

Professor Madhukar Pai, Editor-in-Chief

Julia Robinson, Executive Editor

Re: Resubmission of manuscript, “Nutritional Care for Children with Feeding Difficulties and Disabilities: A Scoping Review,” for publication in *PLOS Global Public Health*

Dear Dr. Kyobutungi, Professor Pai, and Ms. Robinson,

We are pleased to re-submit an original research article titled “Nutritional Care for Children with Feeding Difficulties and Disabilities: A Scoping Review” for consideration in the journal *PLOS Global Public Health*. We would like to thank the reviewers for the thoughtful comments and suggestions that they made, and have worked to strengthen the manuscript in accordance with their feedback. We have also responded to the editor comments based on journal requirements. We have included a point by point response the editor and reviewer comments in the table below. Please let us know if there are any remaining questions or comments.

| **Page or section** | **Comment** | **Response to Reviewers** |
| --- | --- | --- |
| **Editor Comments** |  |  |
| Journal Requirement | Please send a completed 'Competing Interests' statement, including any COIs declared by your co-authors. If you have no competing interests to declare, please state "The authors have declared that no competing interests exist". Otherwise please declare all competing interests beginning with the statement "I have read the journal's policy and the authors of this manuscript have the following competing interests:" | We have included a Competing Interests statement at the end of the document, following the acknowledgements section. We have revised this to start with the sentence you suggested. The Competing Interests Statement now reads: "I have read the journal's policy and the authors of this manuscript have the following competing interests: CMK has been involved in the implementation and evaluation of the Multi-Agency International Training and Support (MAITS) Working with Infants with Feeding Difficulties and Baby Ubuntu programmatic approaches in Rwanda. The authors have no other conflicts of interest to report." |
| Journal Requirement | Please provide separate figure files in .tif or .eps format. (guidance linked in email) | Thank you, we have updated the figures to meet the file requirements. |
| Journal Requirement | We noticed that you used “data not shown”/"unpublished data" in the manuscript. We do not allow these references, as the PLOS data access policy requires that all data be either published with the manuscript or made available in a publicly accessible database. Please amend the supplementary material to include the referenced data or remove the reference. | Thank you for this clarification. We have revised our language in the methods section to avoid confusion. We have not used any unpublished data, however our review did include grey literature such as non-peer reviewed programmatic reports. |
| Journal Requirement | We have noticed that you have uploaded Supporting Information files, but you have not included a list of legends. Please add a full list of legends for your Supporting Information files after the references list. | Thank you. We have added a list of legends to the end of the document which lists the figure heading as well as the PRISMA checklist, which was uploaded as a supplementary document |
| Journal Requirement | In the online submission form, you indicated that your data will be submitted to a repository upon acceptance. We strongly recommend all authors deposit their data before acceptance, as the process can be lengthy and hold up publication timelines. Please note that, though access restrictions are acceptable now, your entire data will need to be made freely accessible if your manuscript is accepted for publication. This policy applies to all data except where public deposition would breach compliance with the protocol approved by your research ethics board. If you are unable to adhere to our open data policy, please kindly revise your statement to explain your reasoning and we will seek the editor's input on an exemption. Please be assured that, once you have provided your new statement, the assessment of your exemption will not hold up the peer review process. | Our data has been submitted to the USAID Data Development Library, which is an open data repository for USAID-funded work. USAID will review the submission and it will be posted online after it clears that process. Reference number: 435-9 |
| Journal Requirement | Please review your reference list to ensure that it is complete and correct. If you have cited papers that have been retracted, please include the rationale for doing so in the manuscript text, or remove these references and replace them with relevant current references. Any changes to the reference list should be mentioned in the rebuttal letter that accompanies your revised manuscript. If you need to cite a retracted article, indicate the article’s retracted status in the References list and also include a citation and full reference for the retraction notice. | We have reviewed the reference list and have ensured it is complete and correct. |
| Editor Comment: Discussion | The first paragraph of the discussion should contain a summary of the study results and the main findings and then recommendations. | Thank you for this clarification. We have added the following language to the first paragraph of the discussion to summarize the study results and main findings: "While some resources and promising approaches exist, they are not standardized or universally used, staff are not trained to use them, and there is insufficient funding to implement them. The combination of challenges in identifying feeding difficulties, a lack of understanding of the link between disabilities and feeding, and weak or nonexistent referral or specialized services puts these children at risk of malnutrition. Additionally, their families face challenges providing the care they need, including coping with high care demands, accessing support, obtaining appropriate foods, and managing stigma. Based on the findings, we have four areas of recommendations for strengthening the identification of feeding difficulties and service provision to meet the needs of children with feeding difficulties—whether or not they are disabled—and their caregivers. These recommendations are to: (1) build the evidence base, (2) strengthen systems to improve identification and service provision; (3) provide direct support to families; and (4) conduct advocacy." |
| **Reviewer 1 Comments** |  |  |
| Reviewer 1: General | Main area for strengthening: It is inconsistent as to whether it focuses only on feeding difficulties in disabled children, or also covers feeding difficulties in non-disabled children | Thank you for this comment. Our review covered both groups of children, however it can be challenging to parse the two groups out given the high proportion of children with disabilities who have a feeding difficulty. Adhering to the social model of disability, we recognize that it is in fact the systematic barriers and discrimination that lead to disability when they interact with an individual's functional abilities and impairments. We reviewed the entire manuscript with this in mind and used more consistent and clear terms throughout. We also added a sentence in the limitations to acknowledge the challenges in our review of differentiating between these groups and hope that additional edits to the manuscript using the universal progressive model help to improve clarity for the reader.   The additional sentence included in the limitations is as follows: "Our review included children with feeding difficulties who may or may not have a disability; it was at times challenging to differentiate these groups of children given the high rates of feeding difficulties among children with disabilities. However, most recommendations would benefit all these children by making services more capable of responding to feeding difficulties and more inclusive of all children." |
| Reviewer 1: General | Main area for strengthening: the findings need to be presented in a clearer framework. This will make the findings and discussion sections sharper. | We appreciate this feedback. We used the Universal Progressive Model, from the Nurturing Care Framework, to organize our analyses looking at the accessibility and responsiveness of routine services for children with feeding difficulties and disabilities, as well as targeted services to manage feeding difficulties and indicated services for the essential additional support required for children with disabilities and their families. We have explained this model in our methods section and have provided some re-organization of our results to align with this model that we hope improves clarity.   The additional sentence in the methods section, as follows, includes a figure showing the application to the Universal Progressive Model to our review: "The Universal Progressive Model for services outlined in the Nurturing Care Framework (WHO, UNICEF, and the World Bank 2018) was used to organize our findings from universal services for all children up to indicated services for children requiring specialized care with referral processes to move across levels, which aligns with a twin-track approach for ensuring equity for persons with disabilities (Figure 2)." |
| Abstract: Methods | Methods: you state that “findings were organized using structured matrices of challenges and opportunities in the identification, management, and support for children with feeding difficulties, disabilities, and their families”. This is not quite how you structure the findings, however (see later comments) | We appreciate your helpful feedback on how to provide greater clarity of our results to align with the way we organized our analyses process. We have made a number of changes in response to yours and the other reviewers comments that better align how we organize our results section using the Universal Progressive Model. |
| Introduction | You frame this as about disability (first few paragraphs) but then you say (line 87) that you cover non-disability related feeding difficulties; reinforced by line 91 (especially children with disabilities). I'm not sure this is consistent (see later comments). If it is right, you should include a section on non-disability related feeding difficulties – what they are – and reconsider whether the initial overarching framing should be about disability or about feeding difficulties with disability as a secondary theme. | We have tried to improve precision of our language throughout the paper to address this concern. We also hope the reorganization of our findings around the Universal Progressive Model that was used in analysis helps provide greater clarity. In addition, as noted above in response to your general comment, we added language to the limitations to acknowledge the challenge of differentiation of children with feeding difficulties without a disability from children with feeding difficulties and a disability given that the only difference is whether the feeding difficulties, or other functional impairments, interact with societal barriers to create disability. |
| Introduction | Is the figure in line 76/77 just for LMICs, or global? Clarify. | We have clarified that this figure is the "prevalence of feeding difficulties in the **global** pediatric population". |
| Introduction Lines 85-88 | I would clarify that you have only focused this review on LMICs. | We have clarified that the review primarily included literature on LMICs but that "relevant literature from high-income contexts was included since services could be adapted to other contexts." |
| Methods | What is a non-systematic review – can you define and it provide a reference? | Thank you for this question. We used the term non-systematic scoping review to differentiate the methods we used from a more traditional systematic review. This was important for this study given that our goal was to provide a broad overview of the opportunities to improve nutritional care for children with feeding difficulties and disabilities, for which we wanted to include not only peer-reviewed articles that we would be able to identify through a systematic review process but also key informant perspectives and non-peer reviewed materials including reports or programmatic tools. We have provided additional clarifications in both the methods and limitations sections about this methodology, and referenced a helpful article differentiating the use of scoping reviews and systematic reviews.   To the methods section, we have added the following sentence: "The scoping review method was selected over a systematic review given the need to more broadly and flexibly review the literature, and non-peer reviewed literature and tools, on the subject (Munn et al., 2018)."  To the limitations, we have also added the following sentence: "In addition, this was not a systematic review given the goals to more broadly assess the current landscape in terms of both peer-reviewed literature and non-peer reviewed materials; a systematic review of standard databases for peer reviewed literature that includes an assessment of the quality of each study included may be more appropriate in the coming years as hopefully more peer-reviewed evaluations of early intervention and disability-inclusive nutrition programs become available" |
| Methods Line 142 | you have a slightly different framing of how you organize the results than appears in the abstract (prevalence, identification, support to manage). | Thank you for noting this. The revision of the methods to explain how we analyzed the findings using the Universal Progressive Model provides additional clarity that is consistent with the abstract. In addition, we specifically updated the abstract to reference this model used in analysis. |
| Methods | If you have separate matrices for the document review and the KIIs, can you explain how you synthesized across the two? | We added an additional sentence to provide more information about how we synthesized findings using our two matrices. We pulled out the main themes from each matrix, looked for areas of commonality and used the Universal Progressive Model as a framework to organize our findings from the two matrices. |
| Results Figure 1 | I think lines 153-162 are really about methods and should come in your methods section on the desk review (i.e. around line 129) | We moved these lines up into the methods section instead of the results. |
| Results Figure 1 | Explain the rationale and process for dropping the first 31 | We have clarified in Figure 1 that the first 31 resources were excluded because a "review of abstract or summary deemed them irrelevant". |
| Results Figure 1 | You say here (reason 3) that you exclude studies that talk about nutrition/feeding but not disability. This implies that you only include studies that mention disability. But this is inconsistent with the framing above. And also inconsistent with your framing in line 163/4. | We have corrected the language for Reason 3 in Figure 1 to say that studies were excluded if they talked about nutrition or feeding but not feeding difficulties or disability. |
| Results Figure 1 | I think it would be helpful to say how many studies/documents were about challenges facing children, and how many are about actual interventions. This would help you be clearer on the division with promising practices, later on | We have added the following sentence to the description of the resources that accompanies Figure 1: "Twenty-two percent (n=28) of resources were related to programs or specific interventions." |
| Results Document | You need to be clear (line 163) on whether this finding is a conclusion you draw from the search (we didn’t find much robust research) or whether this is a finding that is contained in the documents you reviewed/was mentioned by the KIIs. If it is the former, you will need to define ‘robust’ and ‘quality’ in this context and how you made the judgement. | We have removed the term robust and clarified that this is a finding from documents and KIIs |
| Results Participant Characteristics | I would shift lines 175-180 to the methods section about KIIs | We have moved these lines that discuss the participant characteristics to methods section. |
| Results Health Systems | To be honest, I struggled a little with the structure by which you’ve presented the results. | We appreciate this comment, as well as your other related comments, and hope the reorganization around the Universal Progressive Model improves the clarity of how we presented the results. |
| Results Health Systems | Table 2 is like a summary table of how the findings relate to the WHO building blocks – but you haven’t introduced the concept of the building blocks (or said up front you will analyze the data this way). You don’t use this framing in the subsequent text. I did wonder if you would be better off moving this to the discussion as a way of pulling together some of the findings when you make your recommendation 2. If you moved it there, this would add value. At the moment it confuses the narrative. | The table is moved to recommendations, where suggested, and we ensured that no new findings emerge in the table. |
| Results Health Systems | You then structure by identification and then management, and then have a completely new section for communities/family. This is slightly different again from all of your previous framings. | Thank you for this comment. We have reorganized our findings to align with the Universal Progressive Model, which was used for analysis and introduced in our methods section (new Figure 2) based on your prior comments. We also clarified that alongside this model, we specifically considered the enabling environment in terms of community and families to be able to benefit from available services which was a significant consideration in our results. The reorganization we hope improves clarity and a paragraph that referred to a specific level of service for families was moved to the section on indicated services in alignment with the reorganization around the Universal Progressive Model. |
| Results Identification | Table 3 includes some tools that are specific to the disabled and some that are not. Whether this is a problem will depend on the earlier issue of being precise on what you are covering. | We added language to this section to clarify this point. It now includes "The tools in this table either address the identification of feeding difficulties alone, or the bigger nexus of identifying feeding difficulties among children with disabilities (sometimes within broader support for children with disabilities and their families and sometimes not)." |
| Results Identification | In the text, you should have a para on the evidence base (that is included in the final column on the table) about the evidence on the potential impact of using the tools. Including the limitations of the evidence. | We have added additional text to several sections of the paper to respond to this comment.   The following text was added to the section on identification in universal services: "The tools in this table either address the identification of feeding difficulties alone, or the bigger nexus of identifying feeding difficulties among children with disabilities (sometimes within more indicated support services for children with disabilities and their families and sometimes not). For example, the EDACS was identified as a valid and reliable system for use in clinical and research contexts (17)."   The following text was added to the section on targeted support: "This review found tools that support health workers in providing services to address feeding difficulties (see table 2, above). Some of these tools have been evaluated and shown positive results associated with their use though often only in small scale, observational studies. For example, observational studies have shown decrease in feeding difficulties [21], decreased anemia and wasting [23], increased early breastfeeding and exclusive breastfeeding in neonatal care units [27], as well as family benefits such as to improved mealtime cooperation and caregiver stress [29]. While these are positive outcomes, a number of these evaluations did not find improvements in child growth. Additionally, not all tools have yet been evaluated, and additional evaluations would provide useful information about how to adapt, scale and use these and other tools in various contexts." |
| Results Identification | I’m just noting that all of the content in this section is really about challenges in the tools being used as designed, but you state elsewhere that you also want to include promising practices (which are not mentioned here, except for the evidence on the tools themselves). | Thank you. In addition to the paragraph above, we have added the following sentence to our results to complement the promising practices in the use of specific tools. The new sentence reads: "Examples of a promising practice for strengthening government systems includes building a pipeline of speech and language therapists through a local university training program in Ghana, and working with government to overcome barriers to integration of this new workforce into routine services (Interview with a SLT); integration of follow-up care for small and sick newborns including specialized feeding support in Rwanda (Interview with a nurse); and working with community health workers to help facilitate identification and provide initial support services (Interviews with a pediatrician, occupational therapist, physiotherapist, and psychologist)." |
| Results Identification | If there are no promising practices aside from the tools in the table (e.g. no evidence on complementary system strengthening interventions to drive uptake of tools) then you need to say so explicitly. This comment applies to the next section also. | Thank you. We have added additional information on other promising approaches as noted in the above response. |
| Results Services | A couple of typos (e.g. line 257) | We have corrected this error (changed from "LMIC ," to "LMICs,"), and we ran a spell check to ensure there were no other errors in the paper. |
| Results Services | I don’t think (umbrella comment) adding dates for all of the interviews in brackets is necessary – it doesn’t add anything and undermines readability. | We have removed the interview dates throughout. |
| Results Services | These are also all about problems, and nothing about interventions to overcome them | We have added specific reference to several examples on training speech and language therapists, expanding available follow-up care for small and sick newborns, and linking with community health worker programs as specific approaches that show promise. The exact sentence added was mentioned in an earlier comment. |
| Results Communities | The first three sentences are inconsistent about whether they are talking about generic feeding difficulties, or only for disabled. This is an example where more precision would help (this is an overarching comment) | Thank you. We have tried to provide more precision throughout the manuscript in response to this and other related comments. For this specific section, we have re-organized two paragraphs. The first one focuses specifically on feeding difficulties and family needs, such as specific foods and adaptive equipment. All references to additional needs of children with disabilities with feeding difficulties now follow in a separate paragraph. |
| Results Communities | Only in this section do you have examples of promising practices | In addition to promising practices of peer support groups that were already included in the paper, we added additional text about other promising approaches for strengthening health systems as noted in comments above. Also, we moved the promising practices about peer support groups that was originally in the communities section up to the section on indicated services as part of the reorganization using the Universal Progressive Model. We feel these changes improve the clarity of the paper and are grateful for your feedback. |
| Discussion | You should sharpen this based on the comments above (e.g., the fact that you have identified no examples of system level interventions to drive the uptake of tools) | We have revised the discussion to respond to your comments. However, we have highlighted additional promising practices identified in our review that were not well featured in the first draft. The discussion section on strengthening systems has been revised, and includes the following text to adequately reflect both the gaps and promising practices: "Next, strengthened health systems are needed to improve identification and service provision for children with feeding difficulties in health services at all levels of the universal progressive model. This scoping review revealed gaps across all six WHO health systems building blocks for children with feeding difficulties and disabilities (table 3) [59). Promising practices identified in the review were primarily focused on strengthening service delivery, such as capacity strengthening and workforce development. However, this study focused primarily on service delivery and human resources and additional research into the other building blocks is warranted. " |
| Discussion | On the first recommendation, you may wish to be clear that there needs to be evidence generated on i) the efficacy and effectiveness of tools for identification and management, and ii) interventions that overcome the barriers to the uptake of these tools | Thank you for this suggested language. We added the following sentence to the discussion section in the paragraph describing the first recommendation. "Additionally, there needs to be more evidence generated on i) the efficacy and effectiveness of tools for identification and management of feeding difficulties, and ii) interventions that overcome the barriers to the uptake of these tools." |
| Discussion | On the second recommendation, I would bring your table etc. from the start of the results here, it would flow nicely | The table on health systems building block gaps has been moved to the discussion. We agree that this placement is a more appropriate place to discuss the building blocks as this was not the framework we used for analysis but is helpful for interpretation of the results. |
| Limitations | It would be good to reflect on the limits of doing a non-systematic review | Thank you for raising this. As we noted above when we provided additional clarifications on the scoping review method, we have added an additional sentence to the limitations about this as follows: "In addition, this was not a systematic review given the goals to more broadly assess the current landscape in terms of both peer-reviewed literature and non-peer reviewed materials; a systematic review of standard databases for peer reviewed literature that includes an assessment of the quality of each study included may be more appropriate in the coming years as hopefully more peer-reviewed evaluations of early intervention and disability-inclusive nutrition programs become available." |
| Conclusion | This is likely to need some tweaking based on the edits above. | We have updated the conclusion to align with the edits above. The conclusion now reads: "This scoping review found insufficient support for children with feeding difficulties and their families. While some resources and promising system strengthening approaches to identify and support these children exist, they are not standardized or universally used and not enough funding is available to implement them. The combination of challenges in identifying children with feeding difficulties and disabilities, and providing targeted and indicated services for them puts them at risk of malnutrition. Additionally, their families face challenges accessing support and providing the care they need, including coping with high care demands, obtaining appropriate foods, and overcoming stigma for children with disabilities. Four areas of recommendations emerged from this work: (1) build the evidence base for effective interventions to identify and support these children and their families; (2) strengthen health systems to improve identification and service provision, and expand the availability of feeding services; (3) provide direct support to families to address determinants that affect nutrition outcomes; and (4) conduct advocacy to raise awareness of the needs and opportunities related to children with feeding difficulties and disabilities." |
| **Reviewer 2 Comments** |  |  |
| Reviewer 2 General | This is a well-written manuscript addressing programs supporting nutritional care of children with disability and non-disability related feeding difficulties. I congratulate the author(s) to this attempt, as this is an important and understudied topic. Moreover, it is really challenging to conduct a scoping review on this topic, since selecting literature search terms that will thoroughly capture relevant research articles on this topic is not entirely straightforward. Though the authors has prepared the manuscript comprehensively and makes a moderately useful contribution to the subject, it also has scope for improvement in the definition of its scope and goals, in the description of the methods which is incomplete, I feel within this manuscript. | Thank you for this positive feedback as well as your helpful comments to improve our manuscript. |
| Reviewer 2 abstract | The abstract has not followed standard/recommended structure, and scoping review period (Dec 2020-May 2022) and abbreviations, needs to spell out on first use (viz., LMICs, USAID) | We have revised the abstract to include the publication dates of the literature included in the review ("127 documents, ranging in publication date from 2003 to 2022,..."), and we spelled out LMICs and USAID upon first use. |
| Reviewer 2 Methods | Though the objective of the review is clear and the scoping review methodology used is appropriate, the methods section does not provide enough information to know exactly the review work to identify peer-reviewed articles conducted (to identify 187 potential resources) using generic web searches (Google Scholar) without using electronic databases (PubMed, Medline, EMbase, Cochrane Library, Web of Science etc.) and at least a brief summary of the approach is needed. | Thank you for this question and acknowledgement of the appropriateness of our methods approach. We have added additional language to the methods section to provide additional context on our non-systematic scoping review approach per additional comments from Reviewer 1 as explained above. We used a broader search approach in Google Scholar to include articles and resources that may not otherwise appear in electronic databases that are limited to peer reviewed articles.   In the methods section, we have clarified our approach as follows: "The study team used these terms to search for relevant peer reviewed articles, gray literature and programmatic resources. Titles of all identified resources were screened by the review team and only relevant titles were included for a full document review." In addition, we added the following sentence for clarity on how we requested additional resources through working groups and key informants: "We also requested resources from working groups focused on early childhood development, health and nutrition by sending an email inviting group members to share resources directly with the study team and all resources shared were reviewed by the study team." |
| Reviewer 2 General | the authors suggested to revisit the literature search strategy, to explore the existing literature further, and assess whether additional evidence would be pulled with additional targeted search terms, and consider revising the study to re-run the search and add additional evidence to the analysis. | We appreciate this comment and recognize that there would be great value in the future of conducting a systematic review. At this time, we are not able to repeat our full scoping review, but feel the methods used were appropriate to achieve our objective and provide a contribution to the existing literature at this time.   In addition, our scoping review at this time highlights a major gap on the availability of peer-reviewed articles on interventions to improve nutritional care for children with feeding difficulties and disabilities. However we also saw an increase in resources published in the last five years which shows promise of more attention to these issues. Given this, we have included the following sentence in our limitation section: "In addition, this was not a systematic review given the goals to more broadly assess the current landscape in terms of both peer-reviewed literature and non-peer reviewed materials; a systematic review of standard databases for peer reviewed literature that includes an assessment of the quality of each study included may be more appropriate in the coming years as hopefully more peer-reviewed evaluations of early intervention and disability-inclusive nutrition programs become available." |
| Reviewer 2 General | the quality of studies needs to be further assessed since the studies considered are for short period | We have clarified in the methods that while there was not specific date review guiding our search, the included studies were published between 2003 and 2022. The short period the reviewer was referring to was the period of time that our desk review was conducted. In addition, we clarified that the types of literature included was not only peer review publications but also non peer reviewed studies and other articles, gray literature and programmatic resources. This was also complemented by key informant interviews. The non-systematic scoping review methodology was specifically chosen to allow us to assess this issue from a broad lens. |
| Reviewer 2 General | In conclusion, the subject addressed in this manuscript is worth of investigation and recommend for possible consideration as this is an important contribution to the literature, and the findings provide interesting and important knowledge to the research and practice community. However, before taking a decision for possible consideration of publication, the manuscript needs minor revision on its structure, justification on data sources, results and completeness. | Thank you. We hope you will be pleased with the changes made to the manuscript during this revision. Your comments as well as the other reviewer provided great suggestions that we have integrated in order to improve the clarity of the manuscript. |

Thank you again and we look forward to hearing from you.

Sincerely,

Alyssa Klein

Technical Advisor

USAID Advancing Nutrition

alyssa_klein@jsi.com
